# Development and validation of a prognostic nomogram for early stage non-small cell lung cancer: a study based on the SEER database and a Chinese cohort

**DOI:** 10.1186/s12885-022-10067-8

**Published:** 2022-09-14

**Authors:** Liang Zhou, Yahui Zhang, Wenyu Chen, Niu Niu, Junjie Zhao, Weibo Qi, Yufen Xu

**Affiliations:** 1 Bengbu Medical College Graduate Department, Bengbu, 233000 China; 2 Department of Cardio-Thoracic Surgery, Affiliated Hospital of Jiaxing University, 314000 Zhejiang, People’s Republic of China; 3 Departement of Respiratory, Affiliated Hospital of Jiaxing University, 314000 Zhejiang, People’s Republic of China; 4 Department of Medical Oncology, Affiliated Hospital of Jiaxing University, 314000 Zhejiang, People’s Republic of China

**Keywords:** Non-small cell lung cancer, Nomogram, Overall survival, Stage I and II

## Abstract

**Objective:**

This study aimed to construct a nomogram to effectively predict the overall survival (OS) of patients with early-stage non-small-cell lung cancer (NSCLC).

**Methods:**

For the training and internal validation cohorts, a total of 26,941 patients with stage I and II NSCLC were obtained from the Surveillance, Epidemiology, and End Results (SEER) database. A nomogram was constructed based on the risk factors affecting prognosis using a Cox proportional hazards regression model. And 505 patients were recruited from Jiaxing First Hospital for external validation. The discrimination and calibration of the nomogram were evaluated by C-index and calibration curves.

**Results:**

A Nomogram was created after identifying independent prognostic factors using univariate and multifactorial factor analysis. The C-index of this nomogram was 0.726 (95% CI, 0.718–0.735) and 0.721 (95% CI, 0.709–0.734) in the training cohort and the internal validation cohort, respectively, and 0.758 (95% CI, 0.691–0.825) in the external validation cohort, which indicates that the model has good discrimination. Calibration curves for 1-, 3-, and 5-year OS probabilities showed good agreement between predicted and actual survival. In addition, DCA analysis showed that the net benefit of the new model was significantly higher than that of the TNM staging system.

**Conclusion:**

We developed and validated a survival prediction model for patients with non-small cell lung cancer in the early stages. This new nomogram is superior to the traditional TNM staging system and can guide clinicians to make the best clinical decisions.

**Supplementary Information:**

The online version contains supplementary material available at 10.1186/s12885-022-10067-8.

## Introduction

Currently, lung cancer is the leading cause of cancer-related deaths worldwide, and it is the leading cause of cancer-related deaths in China in terms of incidence and mortality from malignancies [1]. Lung cancer incidence continues to rise worldwide as a result of increased industrialization and increased access to tobacco, making lung cancer treatment a critical health issue [2]. Approximately 85% of lung cancer is non-small-cell lung cancer (NSCLC) [3]. Because the early disease is typically asymptomatic, up to 61% of patients have progressed to an advanced stage at the time of diagnosis, which has an inferior prognosis with a five-year survival rate of 18% [4, 5]. However, the prognosis of patients with early-stage lung cancer has a 5-year relative survival rate > 80% [6]. Surgical treatment remains the current treatment of choice for patients with early-stage NSCLC. In clinical practice, the TNM staging method, which is based on the extent of the primary tumor, regional lymph node involvement, and distant metastases, is widely used to predict the prognosis of lung cancer [7]. However, at the same stage, the survival rate of patients varies substantially [8, 9]. This implies that other factors impact the prognosis of NSCLC patients. Clinical characteristics such as gender, age, histology, cell differentiation, the number of lymph nodes examined, distal metastasis, treatment modality (including surgical procedure), chemotherapy (including regimen and cycle), and radiotherapy sequence, for example, are all factors that influence individual cancer patients' survival outcomes [10, 11].

Nomograms are currently regarded as a credible method for quantifying cancer risk and are commonly utilized in clinical studies. It is a graphical computational technique for predicting the prognosis of tumors by integrating important pathological and clinical features [12, 13]. However, nomograms predicting prognosis and guiding postoperative chemotherapy are rare in early-stage non-small cell lung cancer.

Therefore, in the present study, we built and validated the nomogram combined with several clinical variables to predict the prognosis for patients with early-stage NSCLC. In addition, this model is also validated by a unique external cohort in China. Finally, it is compared with the Norman diagram based on the traditional TNM system to evaluate its prediction effectiveness.

## Methods

### Patients and selection criteria

Clinicopathological data and individualized prognostic outcomes in patients with early-stage NSCLC between 2010 and 2015 were obtained from the Surveillance, Epidemiology, and End Results (SEER) database of the National Cancer Institute using SEER*Stat software (version 8.3.9; Incidence – SEER 18 Regs Custom Data (with additional treatment fields), Nov 2018 Sub (1975–2016 varying). The identification of early-stage NSCLC patients was based on the inclusion criteria as follows: (1) confirmed pathology of primary NSCLC; (2) age at diagnosis ≥ 18 years; (3) only patients diagnosed with pathologic stage I or pathologic stage II NSCLC were included. The exclusion criteria were as follows: (1) patients with stage III and above; (2) patients with other primary malignancies; (3) patients who lack information on survival time, metastasis and clinical staging or other incomplete information; and (4) a postoperative survival time < 1 month.

In addition, to test the universality of the model, we reviewed 505 patients with pathological diagnosis of non-small cell lung cancer from January 2015 to December 2017 from Jiaxing First Hospital as an external validation cohort.

There was no requirement for ethical approval since all of the data from the SEER database was obtained in a public method. And the participants in the external validation have been ethically approved by our institution(Ethics No.LS2021-KY-140).

### Study variables

Collect and use the following patient information: Patient characteristics (age, race, sex, vital status, survival time), tumor characteristics (Histological type, tumor size, number of tumors, laterality, primary site, grade of differentiation, AJCC stage, T stage, N stage, number of lymph nodes examined, positive lymph nodes), and Additional treatment (radiotherapy and chemotherapy) and surgical information. According to the SEER code of lung surgery, surgical procedures are classified as Sub-lobectomy, Lobectomy, Pneumonectomy, and Ablation. In the analysis some continuous variables were transformed into categorical variables, such as age, tumor size, and number of lymph nodes cleared, and patients of specific age at diagnosis were classified into four groups (< 50, 50–59, 60–69, ≥ 70 years) according to accepted cut-off values; in this study, the three criteria of T1 (a, b, and c) were classified into (< 10 mm, 10–19 mm, 20–29 mm, and ≥ 30 mm) for tumor size and (0, 1–9, 10–19, 20–29, and ≥ 30) for number of lymph nodes cleared with reference to the eighth edition of the staging system.

### Construction of the nomogram

Using the median, continuous variables such as age and number of cleared lymph nodes were turned into categorical variables. Survival times for categorical variables were compared using the log-rank test in univariate analysis, and survival curves were drawn using the Kaplan–Meier method. The variables with *P* values of < 0.05 were then subjected to multivariate cox regression analysis to screen for risk factors and independent prognostic factors for OS in the training cohort, and hazard ratios (HR) and corresponding 95% confidence intervals (95% Ci) for the variables were calculated. Based on these independent prognostic factors, we used the statistical software (R4.1.2, http://www.rproject.org)) to establish a nomogram to predict the probability of OS rates at 1, 3 and 5 years after radical surgery in patients with early NSCLC.

### Discrimination and calibration of the nomogram

Consistency Index (C-index) and calibration curves are frequently employed to evaluate the performance and accuracy of a nomogram. The C-index values range from 0.5 to 1, and is positively correlated with the predictive performance of the model. When the value is greater than 0.7, the results demonstrated that the model has a reliable discriminant ability [14]. For the verification of the prediction model, the verification queue is utilized for internal verification, and the cases collected by our hospital are used for independent external verification, with bootstrap resampling used to create the calibration curve. The calibration curve is a straight line with a slope of 1 through the origin of the axis. The closer the predicted calibration curve is to the standard curve, the higher the predictive power of the nomogram.

DCA is a novel analytical technique that integrates all clinical consequences of a decision and then quantifies the clinical utility of a predictive model [15]. Furthermore, we employ decision curve analysis (DCA) to determine whether the nomogram is more accurate than the AJCC TNM staging system in order to further assess the benefits and advantages of the nomogram.

## Results

### Study cohort

Twenty-six thousand nine hundred forty-one patients with stage I and II NSCLC from 2010–2015 were extracted from the SEER database; in addition, 505 patients with stage I and II NSCLC from 2015–2017 were included as an external validation cohort from the First Hospital of Jiaxing, China. Patients in the SEER database were randomly divided into the training cohort (*n* = 18,805) and the internal validation cohort (*n* = 8,136) according to the ratio of 7:3. In the training cohort, 8300 (44.14%) males and 10505 (55.86%) females were diagnosed with a median age (67 years), and of these patients, 12034 (63.99%) had adenocarcinoma, 14256 (75.81%) underwent lobectomy, and 3286 (17.47%) received postoperative chemotherapy. In the external validation cohort, 178 (35.25%) male patients and 327 (64.75%) female patients were diagnosed with a median age (60 years), and of these patients, 473 (93.66%) had adenocarcinoma, 358 (70.89%) underwent sublobar resection, and 41 (8.12%) patients (8.12%) underwent postoperative chemotherapy. Table 1 shows the demographic and clinicopathological characteristics of the training and external validation groups.

**Table 1:** Demographics and clinicopathologic characteristics of the training and external validation cohor

	Training cohort	Internal validation cohort	External validation cohort
Characteristics	(*N* = 18,805,n(%)	(*N* = 8136), n(%)	(*N* = 505), n(%)
Age			
<50	1003 (5.33)	399 (4.90)	119 (23.56)
50 ~ 59	3252 (17.29)	1480 (18.19)	129 (25.54)
60 ~ 69	6789 (36.10)	2857 (35.12)	166 (32.87)
≥ 70	7761 (41.27)	3400 (41.79)	91 (18.02)
Sex			
Female	10505 (55.86)	4454 (54.74)	327 (64.75)
Male	8300 (44.14)	3682 (45.26)	178 (35.25)
PrimarySite			
Upper	10973 (58.35)	4737 (58.22)	308 (60.99)
Middle	1159 (6.16)	489 (6.01)	44 (8.71)
Lower	6172 (32.82)	2710 (33.31)	140 (27.72)
Other	501 (2.66)	200 (2.46)	13 (2.57)
TumorType			
Adenocarcinoma	12034 (63.99)	5175 (63.61)	473 (93.66)
Squamous cell carcinoma	4482 (23.83)	1957 (24.05)	25 (4.95)
Adenosquamous carcinoma	418 (2.22)	200 (2.46)	1 (0.20)
Large cell carcinoma	374 (1.99)	161 (1.98)	3 (0.59)
Others	1497 (7.96)	643 (7.90)	3 (0.59)
Grade			
Unknown	1536 (8.17)	652 (8.01)	4 (0.79)
Grade I	3861 (20.53)	1664 (20.45)	307 (60.79)
Grade II	7880 (41.90)	3468 (42.63)	97 (19.21)
Grade III	5288 (28.12)	2266 (27.85)	60 (11.88)
Grade IV	240 (1.28)	86 (1.06)	37 (7.33)
Laterality			
Left	7800 (41.48)	3280 (40.31)	213 (42.18)
Right	11005 (58.52)	4856 (59.69)	292 (57.82)
Stage			
IA	8999 (47.85)	3932 (48.33)	445 (88.12)
IB	5053 (26.87)	2173 (26.71)	42 (8.32)
IIA	2524 (13.42)	1119 (13.75)	10 (1.98)
IIB	2229 (11.85)	912 (11.21)	8 (1.58)
T			
T1	9696 (51.56)	4234 (52.04)	416 (82.38)
T2	7185 (38.21)	3126 (38.42)	61 (12.08)
T3	1924 (10.23)	776 (9.54)	28 (5.54)
N			
N0	16822 (89.45)	7249 (89.10)	490 (97.03)
N1	1983 (10.55)	887 (10.90)	15 (2.97)
Surgery			
Sub-lobectomy	3375 (17.95)	1467 (18.03)	358 (70.89)
Lobectomy	14256 (75.81)	6151 (75.60)	145 (28.71)
Pneumonectomy	1110 (5.90)	479 (5.89)	1 (0.20)
Palliative	64 (0.34)	39 (0.48)	1 (0.20)
Chemotherapy			
No/Unknow	15519 (82.53)	6692 (82.25)	464 (91.88)
Yes	3286 (17.47)	1444 (17.75)	41 (8.12)
Nodes			
0	1663 (8.84)	747 (9.18)	97 (19.21)
1 ~ 9	9743 (51.81)	4198 (51.60)	206 (40.79)
10 ~ 19	5380 (28.61)	2287 (28.11)	140 (27.72)
20 ~ 29	1479 (7.86)	609 (7.49)	49 (9.70)
≥ 30	540 (2.87)	295 (3.63)	13 (2.57)
Positive			
No/Unknow	16876 (89.74)	7275 (89.42)	493 (97.62)
Yes	1929 (10.26)	861 (10.58)	12 (2.38)
TumorSize			
≤ 9 mm	791 (4.21)	336 (4.13)	233 (46.14)
10 ~ 19 mm	5707 (30.35)	2490 (30.60)	182 (36.04)
20 ~ 29 mm	5342 (28.41)	2294 (28.20)	51 (10.10)
≥ 30 mm	6965 (37.04)	3016 (37.07)	39 (7.72)
Number			
1	16,053 (85.37)	6968 (85.64)	467 (92.48)
≥ 2	2752 (14.63)	1168 (14.36)	38 (7.52)

### Independent prognostic factors in the training cohort

Univariate analysis showed that tumor Laterality (*p* > 0.005) and tumor number (*p* > 0.005) were not independent prognostic factors, but age, sex, histological type, tumor size, tumor number, anatomical site, degree of differentiation, AJCC stage, number of examined lymph nodes, positive lymph nodes, chemotherapy and type of operation may be prognostic factors affecting OS (*P* < 0.05). Following univariate analysis, a multifactorial Cox regression analysis was performed using the Farword: LR method and the results revealed that they were all strongly associated with patient survival prognosis (*P* < 0.05). The results of the univariate and multifactorial analyses are shown in Table 2.

**Table 2:** Selected factors in the training cohort for building the model by univariate and multivariate Cox regression analysis

Characteristics	Univariate analysis	*P* value	Multivariate analysis	*P* value
	HR (95% CI)		HR (95% CI)	
Age				
<50	Reference		Reference	
50 ~ 59	0.352(0.297 ~ 0.417)	< 0.001	1.279(1.065 ~ 1.535)	< 0.001
60 ~ 69	0.589(0.541 ~ 0.641)	< 0.001	1.482(1.245 ~ 1.763)	0.008
≥ 70	0.724(0.681 ~ 0.77)	< 0.001	1.999(1.681 ~ 2.377)	< 0.001
Sex				
Female	Reference		Reference	
Male	0.602(0.57 ~ 0.637)	< 0.001	1.368(1.292 ~ 1.448)	< 0.001
TumorType				
Adenocarcinoma	Reference		Reference	
Squamous cell carcinoma	4.559(3.661 ~ 5.678)	< 0.001	1.13(1.06 ~ 1.205)	< 0.001
Adenosquamous carcinoma	7.951(6.371 ~ 9.924)	< 0.001	1.227(1.051 ~ 1.432)	< 0.001
Large cell carcinoma	8.139(6.258 ~ 10.586)	< 0.001	1.494(1.263 ~ 1.767)	< 0.001
Others	9.402(7.228 ~ 12.231)	< 0.001	0.312(0.248 ~ 0.392)	< 0.001
Laterality				
Left	Reference			
Right	1.042(0.986 ~ 1.102)	0.147		
Primary site				
Upper lobe	Reference		Reference	
Middle lobe	0.766(0.654 ~ 0.896)	< 0.001	1.016(0.892 ~ 1.158)	< 0.001
Lower lobe	0.593(0.487 ~ 0.722)	< 0.001	1.122(1.056 ~ 1.192)	0.807
Others	0.802(0.683 ~ 0.942)	< 0.001	1.156(0.983 ~ 1.358)	< 0.001
Grade				
Unknown	Reference		Reference	
I	0.477(0.377 ~ 0.604)	< 0.001	0.622(0.533 ~ 0.725)	< 0.001
II	0.269(0.215 ~ 0.336)	< 0.001	1.142(1.001 ~ 1.303)	< 0.001
III	0.7(0.569 ~ 0.862)	< 0.001	1.488(1.304 ~ 1.698)	< 0.001
IV	1.106(0.898 ~ 1.361)	< 0.001	1.261(0.982 ~ 1.618)	< 0.001
Stage				
IA	Reference		Reference	
IB	0.281(0.259 ~ 0.304)	< 0.001	1.47(1.243 ~ 1.739)	< 0.001
IIA	0.539(0.498 ~ 0.584)	< 0.001	1.977(1.641 ~ 2.382)	< 0.001
IIB	0.896(0.823 ~ 0.975)	< 0.001	2.088(1.596 ~ 2.733)	< 0.001
Surgery				
Sub-lobectomy	Reference		Reference	
Lobectomy	0.399(0.292 ~ 0.546)	< 0.001	0.763(0.699 ~ 0.832)	< 0.001
Pneumonectomy	0.371(0.273 ~ 0.505)	< 0.001	0.982(0.865 ~ 1.114)	< 0.001
Palliative	0.716(0.52 ~ 0.984)	< 0.001	1.561(1.131 ~ 2.154)	0.775
Chemotherapy				
No/unknown	Reference		Reference	
Yes	0.568(0.534 ~ 0.605)	< 0.001	0.858(0.795 ~ 0.925)	< 0.001
Nodes				
0	Reference		Reference	
1 ~ 9	1.092(0.919 ~ 1.297)	< 0.001	0.628(0.566 ~ 0.698)	< 0.001
10 ~ 19	0.817(0.698 ~ 0.956)	< 0.001	0.533(0.475 ~ 0.599)	< 0.001
20 ~ 29	0.793(0.675 ~ 0.931)	< 0.001	0.449(0.387 ~ 0.521)	< 0.001
≥ 30	0.772(0.641 ~ 0.93)	< 0.001	0.542(0.451 ~ 0.652)	< 0.001
Positive				
No/unknown	Reference		Reference	
Yes	0.457(0.426 ~ 0.491)	< 0.001	1.339(1.165 ~ 1.539)	< 0.001
TumorSize				
≤ 9 mm	Reference		Reference	
10–19 mm	0.262(0.217 ~ 0.317)	< 0.001	1.289(1.058 ~ 1.572)	< 0.001
20–29 mm	0.392(0.364 ~ 0.421)	< 0.001	1.662(1.363 ~ 2.027)	0.012
≥ 30 mm	0.57(0.533 ~ 0.61)	< 0.001	2.207(1.797 ~ 2.712)	< 0.001
Number				
1	Reference			
≥ 2	0.93(0.864 ~ 1.001)	0.052		

### Prognostic nomogram for os

**Fig. 1:**
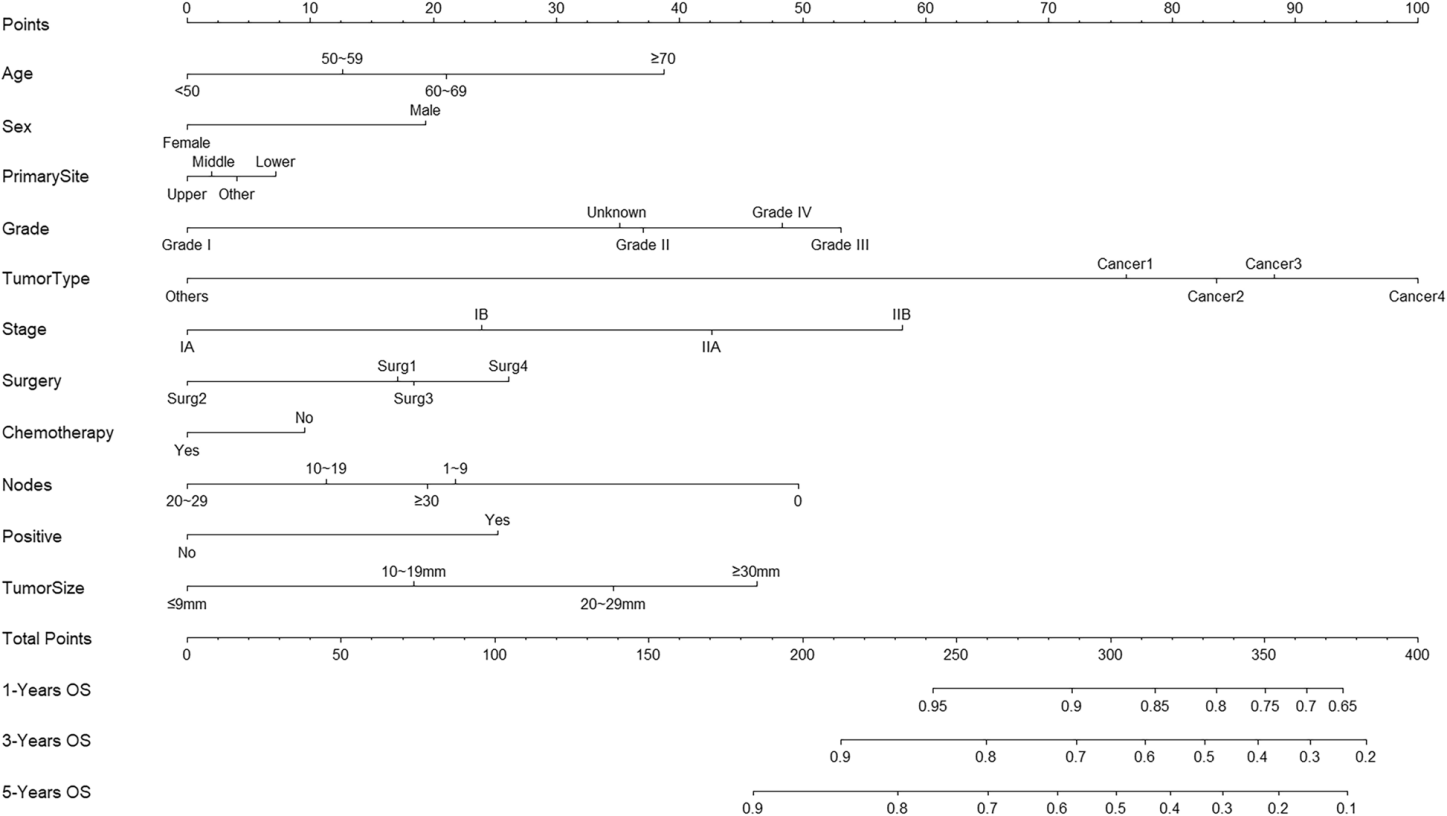
Prognostic nomograms of 1-, 3-, and 5-year OS

According to the results of COX multivariate analysis, 11 independent risk factors, such as age, sex, histological type, tumor size, anatomical site, degree of differentiation, AJCC stage, number of lymph nodes, positive lymph nodes, Chemotherapy and type of surgery, were integrated to create the nomogram (Fig. 1). The probability of survival at 1, 3, and 5 years was easily calculated by summing the scores for each variable to compute the individual risk score and then finding the corresponding point on the survival scale.

### Calibration and validation of the nomogram

**Fig. 2:**
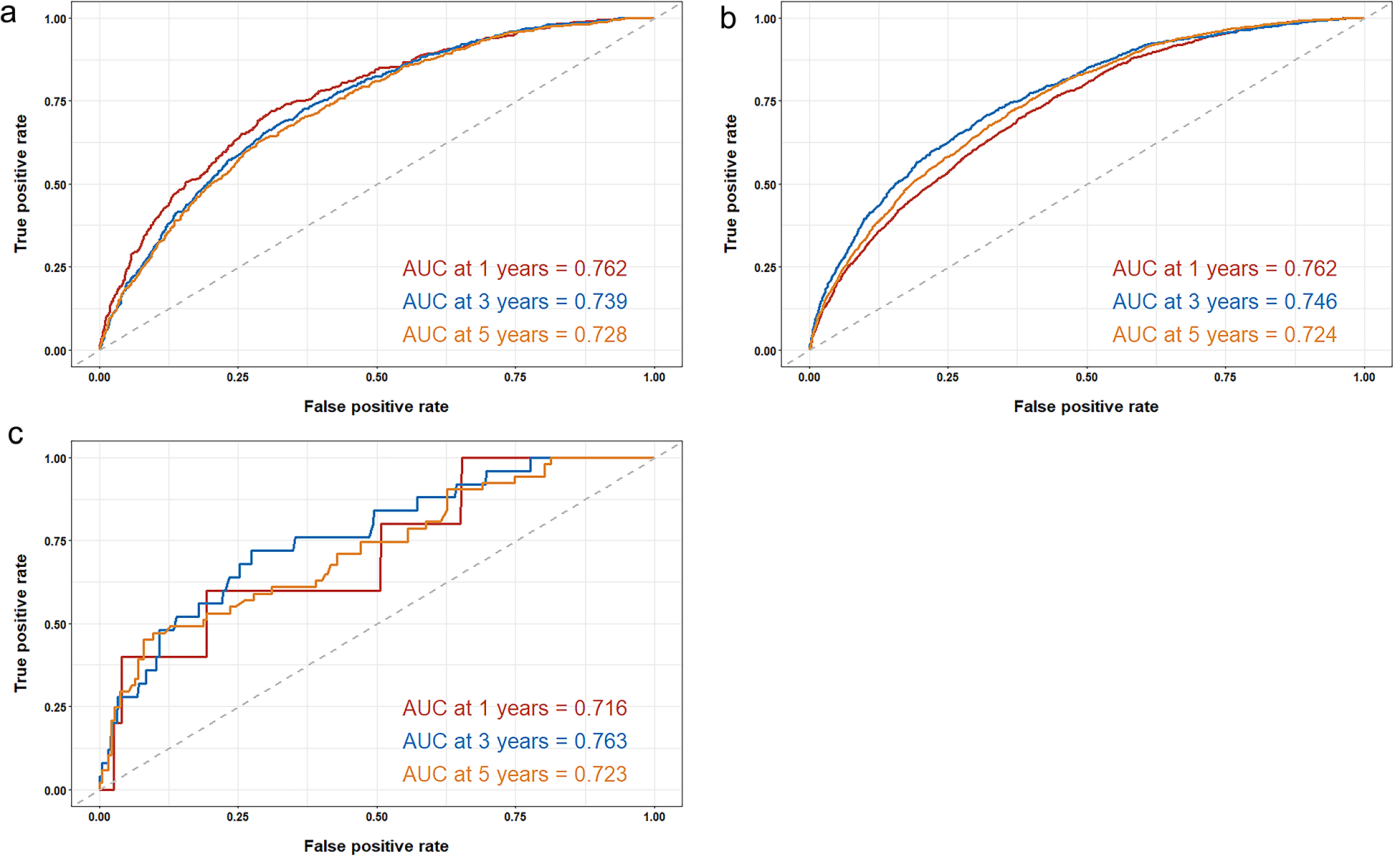
ROC curves and AUCs at 1, 3, and 5 years in the training cohort (**a**) 、internal validation (**b**) and the external validation cohort (c) were used to estimate the prognostic accuracy of the nomogram

**Fig. 3:**
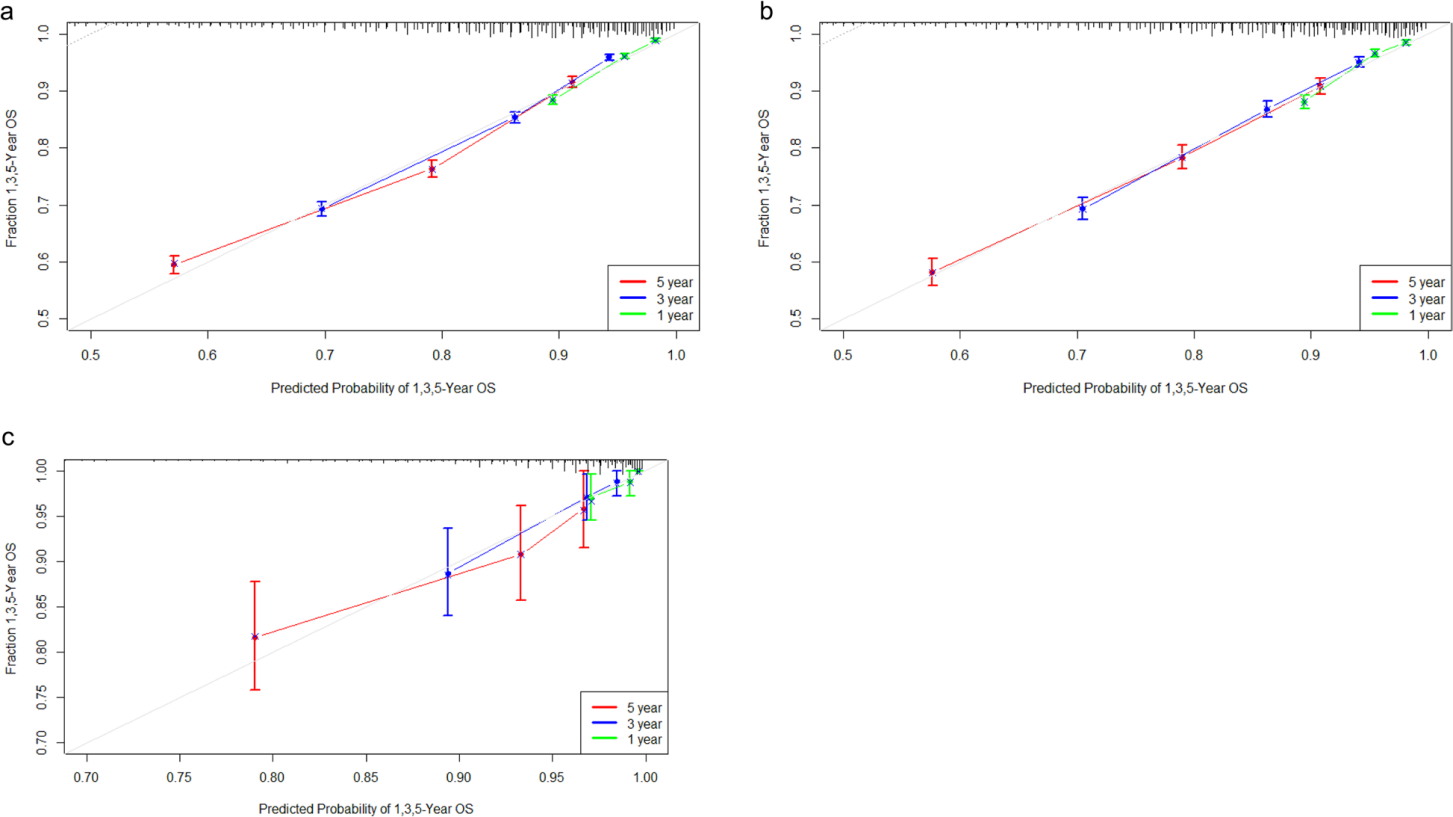
Calibration curves predicting the 1-, 3-, and 5-year OS of patients in the training cohort (**a**) the internal validation cohort (**b**) and the external validation cohort (**c**). The x-axis indicates the predicted survival probability, and the y axis indicates the actual survival probability. The 45-degree line (gray line) indicates that the prediction agrees with actuality

**Fig. 4:**
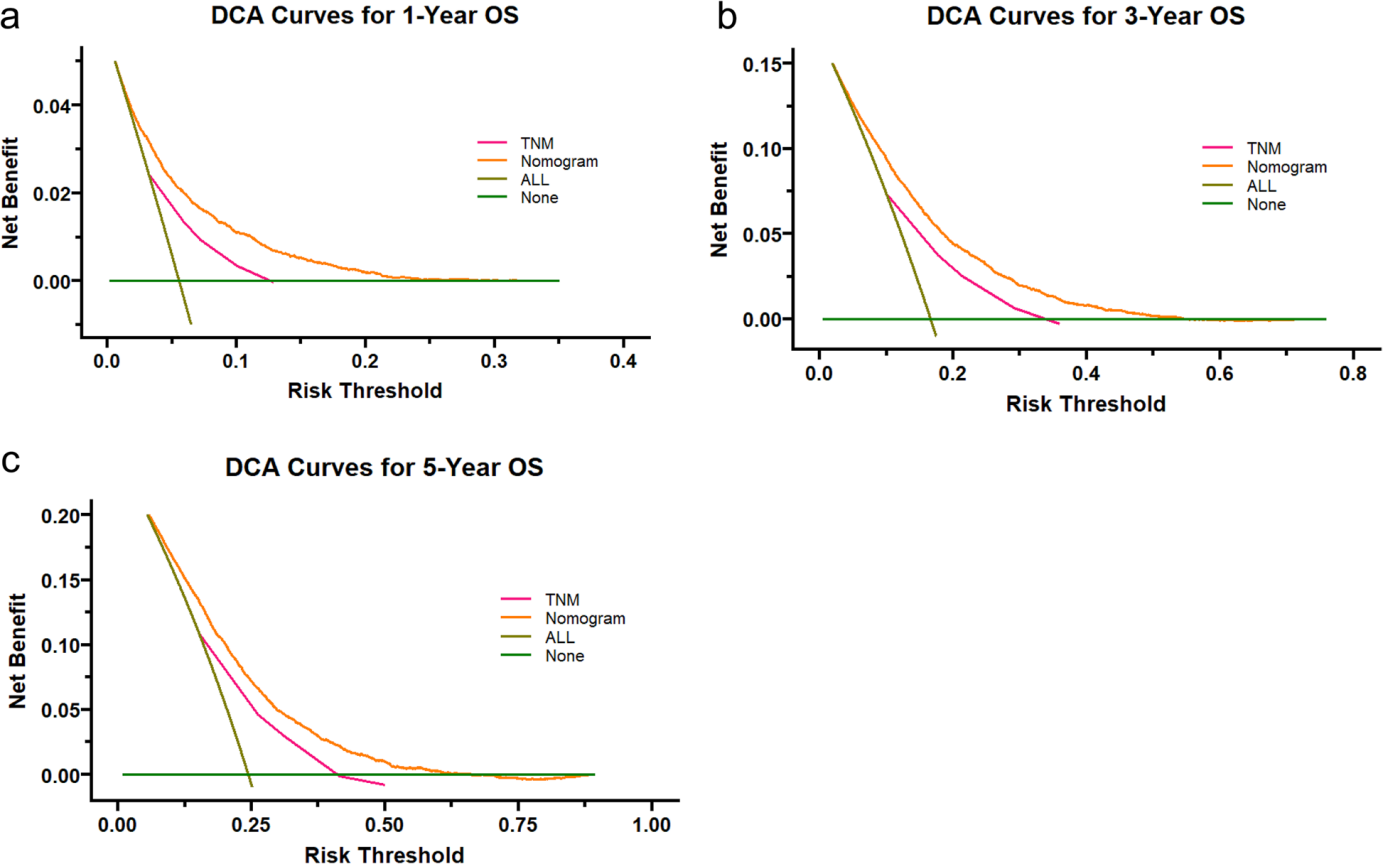
Decision curve analyses (DCA) of the nomogram and AJCC TNM staging system for 1-year (**a**), 3-year (**b**), and 5-year (**c**) overall survival. The x-axis represents the threshold probabilities, and the y-axis measures the net benefit. The horizontal line along the x-axis assumes that overall death occurred in no patients, whereas the solid gray line assumes that all patients will have overall death at a specific threshold probability.The Orange dashed line represents the nomogram. The red dashed line represents AJCC TNM staging system

C-index and AUC values were used to evaluate the accuracy and discrimination of the nomogram. In the training set, the C-index of the nomogram for OS was 0.726(95%CI, 0.718–0.735), and the 1-, 3-, and 5-year AUCs were 0762、0.746、0.724, respectively (Fig. 2a). The C-index in the internal validation set was 0.721 (95% CI, 0.709–0.734), and the 1-, 3-, and 5-year AUCs were 0.762, 0.739, and 0.728, respectively (Fig. 2b). In the external Verification set, the C-index was 0.758(95%CI, 0.691 ~ 0.825) and the 1-, 3- and 5-year AUCs were 0.762, 0.746, and 0.724 respectively (Fig. 2c). We used the calibration plots to check the accuracy of the nomogram and found excellent consistency between the nomogram prediction and the actual prognosis for the training set and validation set (Fig. 3). These results revealed that the nomogram exhibits excellent performance in predicting the OS of Early-Stage NSCLC patients. In addition, we compared the model performance of this nomogram with the conventional AJCC TNM staging system. In the training test, the C-index for the new nomogram and clinical staging of TNM was 0.726 (95% CI, 0.718–0.735) and 0.682 (95% CI: 0.673 to 0.691), respectively. When compared to the AJCC TNM staging method, the DCA analysis revealed a significant increase in a net benefit for the new nomogram chart with a wide and practical range of threshold probabilities (Fig. 4).

### Webserver development for the nomogram

To facilitate clinicians' use of our Nomograms, dynamic line graphs are generated using the "DynNom" package of the R software, registering users and publishing web line graphs on shinyapps.io, the online version of the web server can be accessed directly from the following URL: https://early-stage-nsclc.shinyapps.io/DynNomapp/. After entering the predictor variables on the web server, the dynamic column line graphs can easily display the calculated survival probabilities and generate case-related figures, tables and corresponding survival graphs.

It is simple to use and does not require permission or a login password from any clinician.

### Overall survival analysis

**Fig. 5:**
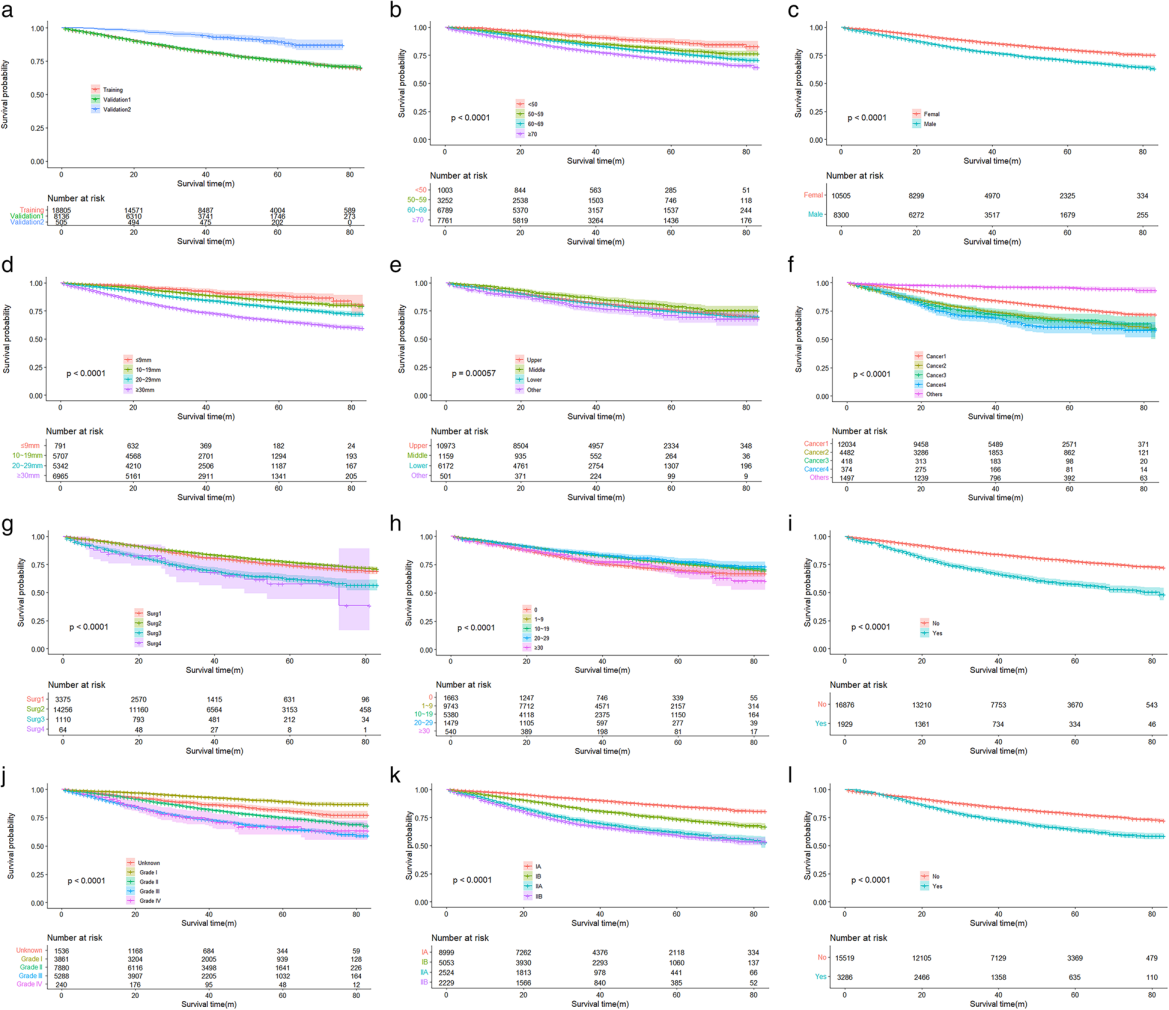
Overall survival rates stratified by patient characteristics. Kaplan–Meier overall survival curves of the training set (*P* < 0.001) according to: (**a**) SEER cohort and China validation cohort; (**b**) age; (**c**) sex; (**c**) tumorsize; (**d**) primary site; (**e**) tumor type; (**f**) surgery; (**g**) nodes; (**h**) positive; (**i**) grade; (**k**) AJCC stage (7th); and (**l**) Chemotherapy

In terms of OS, the China validation cohort outperformed the SEER cohort (Kaplan–Meier curves are shown in Fig. 5). Based on the results of the multifactorial Cox regression analysis, we analyzed the survival curves of patients according to 11 variables. Based on demographic data, the results revealed that OS was considerably lower in older patients (≥ 70 years) than in patients of other ages, and significantly lower in male patients than in female patients. Furthermore, in terms of histologic type, adenocarcinoma has a better prognosis than squamous and large cell carcinoma, while other rare NSCLC subtypes have a really poor prognosis. On the other hand, intermediate and highly differentiated tumors, as well as early AJCC staging, had a better prognosis than poorly differentiated or undifferentiated tumors. Patients who did not undergo in situ resection had a poor prognosis, those who underwent lobectomy had the best prognosis (*P* < 0.001), and those with larger tumors had a poor prognosis. Lymph node dissection is critical for performing the surgical treatment, and those who did not have lymph node dissection had a poor prognosis.

## Discussion

Since surgical resection is still an essential treatment for early-stage NSCLC, the prognosis of postoperative survival is still dependent on the conventional AJCC staging system, which has several limitations, For example, patients with the same stage may have different prognosis, which indicates that it is also closely related to many other independent factors (such as gender, age, tumor histology, degree of tumor differentiation, etc.), not only the tumor size and lymph node involvement in the TNM staging system, so this staging system does not provide clinicians with individualized and more accurate prognosis prediction. Therefore there is a need to establish a well-developed prognostic model to compensate for this limitation. In recent years, many researchers have attempted to build similar survival prediction models, for example, Zuo [16] built a prediction model from the SEER database for patients with stage Ib NSCLC and performed external validation, but the C-index obtained for the training and external validation cohorts was 0.637 (95% CI 0.634–0.641) and 0.667 (95% CI 0.656–0.678). CI 0.656–0.678). The prediction model developed by Zhang with 443 patients with early-stage NSCLC had a C-index of 0.622 (95% CI: 0.572–0.672), although higher than the conventional TNM staging system with a C-index of 0.596 (95% CI. 0.551–0.641), but lacked external validation [17]. The accuracy achieved by the current prediction models developed regarding patients with early-stage NSCLC is not particularly high and therefore difficult to apply in clinical practice. In contrast, this study not only modeled based on a large sample size, but also validated with a large amount of external data, and achieved a high accuracy. In addition, compared to previous models our study attempted to incorporate more parameters to develop a more reliable postoperative predictive nomogram for patients with early-stage NSCLC.

Through univariate and multivariate analyses, we discovered that age, sex, histological type, tumor size, tumor number, anatomical site, degree of differentiation, AJCC stage, number of examined lymph nodes, positive lymph nodes, chemotherapy, and type of surgery were independent factors affecting OS in this large population study, which is consistent with the findings of similar related studies [12, 18]. According to our nomogram, tumor pathological type was the strongest predictor of OS, with large cell lung cancer having the poorest prognosis and adenocarcinoma being the best pathological type. Secondly, tumors with surgical sites occurring in the upper lobes have a relatively good prognosis, and the findings of Li [19] and Lee [20] are consistent with this, suggesting that it may be related to differences in anatomical site, ease of surgical site, degree of lymph node clearance, and adjacent surrounding tissues. It is worth noting that although the anatomy of the left and right sides of the lung differed, the affected side of the tumor (*P* = 0.147) was not a significant independent influencing factor.

The degree of tumor differentiation is closely related to the biological behavior of different types of tumors and therefore naturally affects the prognosis of patients [18]. The findings suggest that the degree of tumor differentiation is positively correlated with the malignancy and aggressiveness of the tumor [21]. However, some scholars disagree that hypodifferentiation is not associated with poorer prognosis in early-stage NSCLC [22]. In our study, poor differentiation was significantly associated with poor survival in early-stage NSCLC, suggesting that this factor may provide useful information for defining the aggressiveness of the tumor. Although the degree of differentiation is now included in the pathological staging of early esophageal cancer [23], it is not included in the TNM staging criteria of lung cancer. Considering that the degree of differentiation can guide surgery and predict survival, we strongly recommend that the degree of differentiation be included in the forthcoming TNM classification criteria.

In early-stage NSCLC, tumor size is an important independent predictor of prognosis. Our findings support the widespread perception that the smaller the tumor, the better the prognosis. The eighth edition of TNM staging of lung cancer divides T1 into three subgroups of a, b, and c on a per centimeter basis, further indicating that tumor size is an extremely important prognostic factor [24]. Our study divided the variables of tumor size according to T1 criteria, which better reflected the survival differences of different tumor sizes compared with previous models. In the training cohort, lobectomy had a better OS, but in the external validation population, where sublobar resection cases were predominant, postoperative survival was not worse than lobectomy. This deserves additional investigation, particularly for patients with stage I NSCLC ≤ 2 cm, where there is no consensus on the best surgical approach. A series of prospective studies on this issue have been conducted in North America (CALGB140503) [25] and Japan (JCOG0802/WJOG4607L) [26], and the latest published results suggest that subpneumonectomy is non-inferior or even slightly superior to lobectomy in early-stage NSCLC [27–31]. Presently, an increasing number of surgical teams are endorsing this outcome and preferring sublobar resection.

The number of lymph nodes removed is an important prognostic factor in various cancers [32, 33], and the thoroughness of lymph node clearance will determine the likelihood of resection of metastatic lymph nodes and lead to accurate staging, which will guide the adjuvant treatment of patients [34]. Similar studies have shown that the higher the number of lymph nodes examined, the better the prognosis [18]. The ACOSOG Z0030 trial, on the other hand, indicated that systemic lymph node dissection no longer improves the oncologic prognosis of early-stage NSCLC if thorough lymph node sampling reveals negative lymph nodes [35]. A study by Wo [36] in patients with stage IA NSCLC showed a decreased survival benefit when more than 10 lymph nodes were examined. In this study, which also included stage II patients, the survival benefit was reduced when the number of lymph nodes cleared exceeded 30. This difference leads us to believe that lymph node dissection should be further investigated depending on the stage, or that the number of stations should be utilized instead of the number.

To minimize overfitting, we verified and calibrated the model, which exhibited reasonably constant discriminative power and calibration curves demonstrating good agreement between predicted survival probabilities and actual data, indicating that the established model is repeatable and reliable. Moreover, the nomogram model has good applicability in the external validation cohort. Besides, the C-index of this nomogram (0.726 (95% Ci, 0.718–0.735)) was higher than that of the conventional TNM staging system (0.682 (95% Ci: 0.673–0.691)), and the DCA curve results demonstrated that the model had greater discriminatory power and clinical utility than the TNM staging system.

However, there are still some limitations of the present study. First, this was a retrospective and non-randomized study subject to all the limitations inherent in the study design. Therefore, prospective studies are also needed to test the validity of this model. Second, there are some limitations to using the SEER database, which only provides crude mortality data and lacks some important covariates, such as smoking history, vascular invasion, lymphovascular invasion, neural invasion, the presence of cancer thrombi, an up-to-date classification of pathological types, genetic mutations, and time to disease progression, as well as specific chemotherapy and targeted therapy, all of which are important prognostic factors in NSCLC [37, 38]. Finally, our Norman plot was created using a large population and validated using external data with good discrimination and consistency, but the external validation data is only for cases in a single region and is not representative of other regions. Hence, more data from different regions is required for external validation. As a result, more multicenter studies and prospective data collection incorporating other potential variables are required to improve this nomogram.

## Conclusions

We have developed and validated a nomogram based on the SEER large population database to provide a convenient and reliable individualized postoperative survival prediction tool for patients with early-stage non-small cell lung cancer.

This new nomogram outperforms the conventional TNM staging system in predicting 1-, 3-, and 5-year survival rates for patients with early-onset non-small cell lung cancer, assisting clinicians in predicting patient prognosis and making treatment decisions. More prospective studies are needed in the future to continuously refine studies related to the survival prognosis of patients with early-stage non-small cell lung cancer.

## Supplementary Information

**Additional file 1.** External verification.

**Additional file 2.** Internal validation.

**Additional file 3.** Training queue.

## Data Availability

The datasets generated and/or analysed during the current study are available in the SEER database (http://seer.cancer.gov) repository. All data generated or analysed during this study are included in this published article and its supplementary information files.
